# R^2^OBBIE-3D, a Fast Robotic High-Resolution System for Quantitative Phenotyping of Surface Geometry and Colour-Texture

**DOI:** 10.1371/journal.pone.0126740

**Published:** 2015-06-03

**Authors:** António F. Martins, Michel Bessant, Liana Manukyan, Michel C. Milinkovitch

**Affiliations:** 1 Laboratory of Artificial & Natural Evolution (LANE), Department of Genetics & Evolution, University of Geneva, Geneva, Switzerland; 2 SIB Swiss Institute of Bioinformatics, Geneva, Switzerland; University of Basel, SWITZERLAND

## Abstract

While recent imaging techniques provide insights into biological processes from the molecular to the cellular scale, phenotypes at larger scales remain poorly amenable to quantitative analyses. For example, investigations of the biophysical mechanisms generating skin morphological complexity and diversity would greatly benefit from 3D geometry and colour-texture reconstructions. Here, we report on R^2^OBBIE-3D, an integrated system that combines a robotic arm, a high-resolution digital colour camera, an illumination basket of high-intensity light-emitting diodes and state-of-the-art 3D-reconstruction approaches. We demonstrate that R^2^OBBIE generates accurate 3D models of biological objects between 1 and 100 cm, makes multiview photometric stereo scanning possible in practical processing times, and enables the capture of colour-texture and geometric resolutions better than 15 μm without the use of magnifying lenses. R^2^OBBIE has the potential to greatly improve quantitative analyses of phenotypes in addition to providing multiple new applications in, *e*.*g*., biomedical science.

## Introduction

For the past decade, major advances in quantitative and systems biology have been driven by the integration of physics and computer science approaches with innovative developments in molecular biology, imaging [1] as well as micro- and nano-manipulations [2]. These new technologies opened the way to investigations of, among others, single-molecule [3] and subcellular dynamics [4], cellular mechanics [2], and tissue growth/homeostasis during development [5, 6]. Hence, most innovative imaging techniques deal with the very small, *i*.*e*., from the molecular scale to the cell scale. Surprisingly, similar breakthroughs for imaging and quantifying phenotypes at larger scales (from a dozen micrometres to the decimetre scale) are rare, hence, have hindered high-throughput and quantitative analyses of morphological phenotypes (with the exception of X-ray computed tomography [7]). Given the recent advances in surface scanning hardware and 3D-reconstruction software developed in the computer graphics community, these techniques have the potential to fill this gap if they can be automated, adapted to phenotyping biological material, and brought to texture and geometric resolutions down to a few dozen microns.

Several methods are available for 3D geometry surface scanning. Contact scanners (such as coordinate measuring machines) can achieve very high accuracy and resolution, often below 5 μm [8]. However, running times for high-resolution scanning are very long (~3mm/min) and scanning is restricted to rigid and robust items as the device may cause damage to delicate objects, making this approach unsuitable for scans of living animals. Triangulation-based scanners using laser illumination can achieve accuracy and resolution between ~16μm and a few hundred μm, depending on the scanner, the scanning distance, and the object being scanned [8]. Nevertheless, the process is too slow for imaging living animals and/or processing series of objects at high resolution. Structured-light technology using pattern codification [9] is a widely used hardware/software solution that can yield accuracy and resolution in the order of 100 μm, although a detailed and rigorous evaluation of performances is still lacking in the literature. When a full 360° reconstruction of the object is needed, most structured-light methods are slow and require several scans from different views, making the whole process tedious and error prone due to the need of merging the scans into a final 3D model. This limitation can be overcome using multiple cameras and projectors, (see *e*.*g*. [10]), but a specialised, and generally not flexible setup is required. In addition to their limitations discussed above, the price of high-resolution devices implementing triangulation-based laser and/or structured-light scanning is generally in the range $60,000–350,000. A third approach to 3D geometry reconstruction is laser time-of-flight technology, but these methods are not adequate for close range scanning and have accuracy and resolution in the order of mm at best [11].

Aside from the methods outlined above, passive (*i*.*e*., without projecting lasers or patterns on the object) non-contact approaches have become accessible. The recent development of high-quality consumer digital cameras led to an increasing interest for Structure from Motion (SFM, see *e*.*g*. [12]) systems which derive the 3D geometry from the texture of pictures [13–15]. This approach is faster than laser and structured light approaches, much more flexible, can be easily scaled up, and can even be adapted to acquisition of data in the field. Web resources for benchmarking [16, 17] have facilitated the development of multiple algorithms in the academic community. These benchmarks show that top SFM algorithms perform well: 3D geometry accuracy is about 300 μm and model completeness is 99% even when using images of only three Megapixels [16]. Moreover, SFM resolution quickly improves with the corresponding increase of camera sensor resolution. Most importantly, SFM methods naturally incorporate high-quality colour texture in the reconstruction process, a distinguishing feature that makes them suitable choices if this information is important for the final 3D model. Indeed, although laser and structured light technologies have integrated components for the simultaneous acquisition of texture and geometry, their main focus is on the latter.

Here, we report on the development of a highly-flexible solution: R^2^OBBIE-3D, a robotic scanner combining an industrial six-axis robotic arm, a high-resolution (36 megapixels) digital single-lens reflex (DSLR) colour camera, a mechanical extension, and an illumination basket with high-intensity light-emitting diodes (LEDs). The system (see Fig A in S1 File and S1 Movie) allows the use of two 3D-reconstruction methods: *(i)* SFM, discussed above, that provides accurate low frequency 3D-position surface geometry and high-resolution colour texture, and *(ii)* Photometric Stereo (PS, [18]) that measures high-frequency surface micro-geometry, in the form of point surface orientation, but generates a low-frequency bias. R^2^OBBIE-3D performs flexible, yet highly repeatable, operations such that combined SFM+PS multiview scans become feasible in practical time, whereas the method is highly tedious, if not impossible, to perform manually. R^2^OBBIE-3D generates exceptionally complete models in real situations (*i*.*e*., scanning whole 360° objects) with colour-texture and geometric resolutions of about 15 microns, without the use of magnifying lenses.

R^2^OBBIE-3D was designed to allow researchers in our laboratory to generate digital 3D models of vertebrates under anaesthesia and investigate biophysical mechanisms at the origin of skin morphological complexity and diversity (see, *e*.*g*. [19]). More generally, the system can be used to perform automated high-resolution 3D colour scans of almost any object between 1 and 100 cm, for greatly improved quantitative analyses and for multiple applications in museology, reverse engineering, biomimicry as well as in forensic and biomedical sciences. R^2^OBBIE-3D stands for *‘Robotic and Reptile-Oriented Biological Beauty 3D-Imaging Equipment’*. Robotics aficionados will have realised that the name of our system is inspired by ‘Robbie’, the title of a famous science fiction short story by the late Isaac Asimov. Hence, R^2^OBBIE-3D is pronounced *‘Robee three D’* (*i*.*e*., *‘rɒbiː θriː diː’* using the Oxford English Dictionary key to pronunciation; http://public.oed.com/how-to-use-the-oed/key-to-pronunciation/).

## Materials and Methods

### Animals

Maintenance and housing of, and experiments on animals performed in this study were approved by the Geneva Canton ethical regulation authority (authorisation 1008/3421/1R) and performed according to Swiss law. Anaesthesia was administered through passive inhalation (using an induction chamber) of sevoflurane. The University of Geneva Ethics Committee approved the entire study.

### The extension mechanical assembly

**Fig 1:**
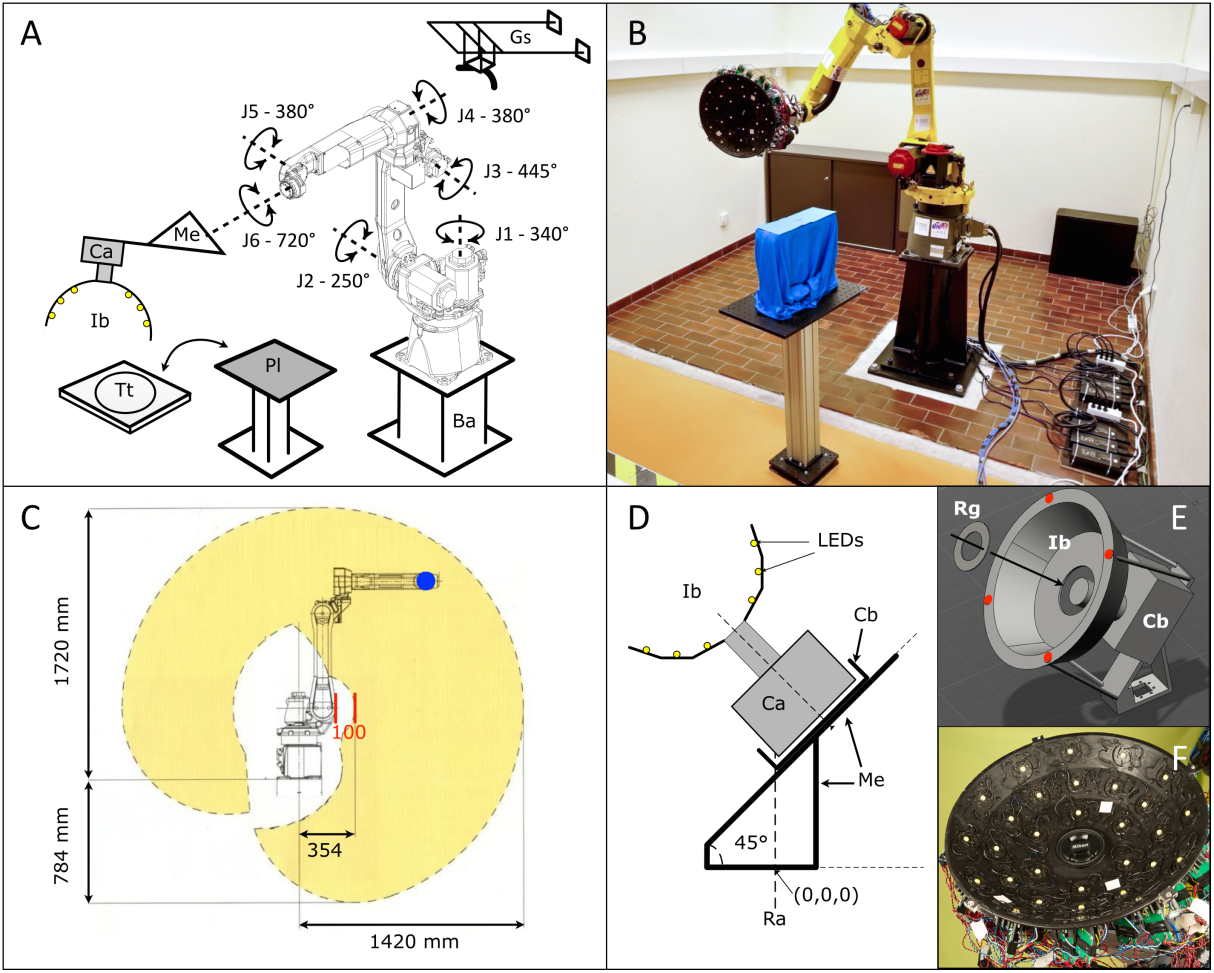
The R^2^OBBIE scanning system. A) Schematic representation of the set up; *J1–J6*, the six axes of the robot; *Ba*, elevated base; *Me*, mechanical extension; *Ca*, camera; *Ib*, illumination basket; *Pl*, platform for positioning the target object; *Tt*, turn-table; *Gs*, gutter to suspend objects. B) R^2^OBBIE with the platform configuration. C) The working envelope (yellow) of the robot J5 axis (blue) in the vertical plane passing by J1. D) Schematic view of the camera (Ca) fixed in the camera box (Cb), the illumination basket (Ib), and the mechanical extension; Ra, Rotation axis of J6. E) The illumination basket is fixed to the camera with a ring (Rg) and by four points (red) to the camera box through cylindrical rods. F) Picture of the illumination basket showing the 30 LEDs in place.

The reach of the robot is maximum 142 cm on the horizontal axis, with a forbidden zone of 10 cm in front of J2 (red bars in Fig 1C). We consider the horizontal plane containing J2 as optimal for placing the target to be scanned, hence, the target platform is positioned at that height. One of our main purposes is to scan <30cm long lizards at distances from 16 to 40 cm from the camera lens. Given the short length of the J5-to-J6 section and the width of the camera body, the J5 axis is largely limited in the amplitude of angles it can reach when the camera box is placed directly on the robot flange (*i*.*e*., without additional mechanical interface). This configuration would greatly reduce the scanning envelop of the system. Hence, we designed and built an aluminium alloy (EN AW-6082) mechanical extension with a 45° angle attached to the robotic arm flange (Fig 1D–1F). The extension increases the reach of the camera, allows larger rotation angles of J5, and removes the 100 mm forbidden zone when the camera is oriented towards J2. Fig A Part A in S1 file shows portions of the scanning envelopes with and without the mechanical extension. The 870 mm scanning envelope available with the mechanical extension allows for scanning 30 cm-long lizards with a 270° coverage at a 40 cm distance. Moreover, simulations performed using COMSOL Multiphysics (v4.3a, Comsol Inc., Burlington, MA, USA) indicate that, even under maximum loading conditions (~75N, corresponding to a deceleration of 10m/s^2^ for the 7.445kg payload of the camera + basket + extension assembly), the maximum von Mises stress exerted on the extension is 13.65 MPa, (*i*.*e*., considerably below the yield stress of the alloy; >110 MPa) and the vibrations do not exceed an amplitude of 0.080 mm (Fig A Part B in S1 file). Using blur metrics, we also show experimentally below (Results section) that vibration attenuation times are short.

### The illumination basket

We built the illumination basket (Fig 1F) by compressing a mix of glass fibres and resin within a custom-made metallic matrix designed to yield the following final geometry: three 6 mm-thick conical sheets connected with angles of 15° (Fig B Part A in S1 file). This shape ensures that an object positioned at 16cm in front of the camera lens (*i*.*e*., the minimal focus distance for the 105mm lens) would be included in the cone of 30° angular displacement of any LED (relative to its normal direction) placed on the internal surface of the basket. This design therefore yields homogenous illumination because it maintains below 10% the loss of intensity due to angular displacement of any LED (Fig B Part B in S1 file). We fixed the 30 LED modules on the basket as follows: six on the most internal cone and twelve on each of the two other cones. For increased rigidity, the illumination basket was fixed to the mechanical extension with four cylindrical rods as well as to the camera lens with a ring (Fig 1E).

Each of the 30 LED modules (Fig B Part C in S1 file) consists of a high-power white LED (2100 lumens, 5500K colour temperature; Led Engin Inc., San Jose, USA) glued with thermal-conductive paste on a high-dissipation heat sink, and two electronic cards including each a constant-current controller (CCC) and an ON/OFF electronic control (OEC). The heat sink provides the necessary security and thermal stability as a LED heats from 25°C to 100°C in about 180s and 30s with and without heat sink, respectively. Considering a SFM scan, we observe that no LED remains ON more than 30% of the time and that all LEDs remain under 65°C indefinitely. Note that *(i)* CCC and LED illumination ensure that light intensity is constant from picture to picture, and *(ii)* the reaction time of this type of electronics is <100ms. Our system is therefore more appropriate than the use of a flash light because the latter requires long charging time and its intensity is difficult to control.

### Electronic controls of camera and LEDs

Optocouplers, as electronic control interfaces for the camera and LEDs, are connected to the digital output of the robot controller to transform +24V digital output signals to ON commands. The advantage of using an optocoupler is to electrically isolate the robot-controller from the device to control and to adapt voltage levels (the two devices using different grounds and different ON voltages). This allows the robot to control directly the focus and trigger of the camera (via the latter’s digital inputs), as well as the ON/OFF state of the LEDs (using the CCCs digital inputs as mediators).

### Robot programming

3D scanning of the target requires defining the successive camera positions and orientations, the camera settings (*e*.*g*., ISO value, f-number, shutter speed), the list of ON/OFF commands for the LEDs and for the camera focusing and triggering, as well as setting up the robot-computer interaction (when required, *e*.*g*., for combined PMVS+PS scans). These positions and commands are compiled into robot language for upload to the robot controller embedded memory. When the program is executed, the robot controller performs the sequence of actions automatically without the need of any manual intervention (Fig C in S1 file). Setting camera positions and orientations requires defining the (X,Y,Z,W,P,R) 3D coordinates, where (X,Y,Z) and (W,P,R) represent the camera position and orientation, respectively. As manual programming of camera positions/orientations on the robot-controller console is time-consuming, inaccurate, and generates suboptimal trajectories, we program these coordinates in MATLAB (v2012a, The MathWorks Inc., Natick, MA, USA) on the basis of two pre-defined concentric parametric surfaces (such as half cylinders or half ellipsoids; Fig D in S1 file), respectively representing the scanning envelope and the idealised object shape. In addition, to improve robustness of SFM 3D reconstruction, images taken at any two nearest-neighbour positions must overlap considerably.

As the trajectories generated with our MATLAB script can correspond to non-reachable positions and singularities (due to collinear alignment of two robot axes, causing unpredictable motion and velocities), we first run the robot program into the *Robotguide* simulation software (Fanuc Corporation). Beside the six-axis robot itself, we introduce in the simulated scene, the mechanical extension, the camera, the illumination basket, the platform/turntable (if any), and the target, such that we can also correct trajectories that generate collisions. Finally, the program is uploaded on the real robot and is validated on the basis of successful final 3D reconstruction. The full programming flow is shown in Fig E in S1 file.

### White-Balance calibration

The acquisition of high-quality, realistic colour texture requires the correct adjustment of the camera’s white balance to the illumination conditions of our setup. Since the LEDs’ spectral power distribution has a complex, bi-modal shape (Fig F Part A in S1 file), standard white balance settings (*e*.*g*., colour temperature) are unable to capture the correct lighting conditions of the scene, resulting in pictures with unrealistic colours (Fig F Part B in S1 file). We therefore calibrated the camera’s white balance to our illumination setup in order to obtain realistic colours (Fig F Part C in S1 file). The generated white balance profile was then embedded in the camera’s internal memory.

### 3D reconstruction software

For SFM, we use ‘Bundler’ [20, 21], which yields estimated camera parameters, as well as a sparse 3D-point-cloud of the scanned object, followed by Patch-Based Multiview Stereo (PMVS, [13, 22]) that produces a dense-point cloud, which is in turn used to generate a mesh model with a Poisson Surface Reconstruction method [23, 24], followed by texture generation through blending the images from different views.

For PS reconstructions, we use the Shape From Shading principle [18]: surface normals are determined by observing the scanned object under multiple different lighting directions and these normals are then integrated to generate the reconstructed surface [25]. We are using 30 non-coplanar light sources to enhance the quality of the recovered normals. The large number of lighting directions, coupled with a pre-processing algorithm to remove shadows and specularities [26, 27], provide efficient noise reduction and the ability to handle regions that deviate from the Lambertian diffuse model assumption. An important feature of PS algorithms is the reconstruction of surface normal for each image pixel. Using our current setup, this allows for meshes with as many as 36 million points (for a single camera position) and a corresponding large number of faces. The PS approach is particularly helpful in: *(i)* obtaining the shape of patternless objects, which are difficult to reconstruct using the SFM pipeline, *(ii)* uncovering geometry and texture of tiny features like scales, sensors, pores, wrinkles, by using high-frequency diffuse normals information [28, 29]. In addition to Structure-From-Motion and Shape-From-Shading methods, R^2^OBBIE can be adapted to use any other image-based method such as, *e*.*g*., Visual Hull [30].

### Experimental setup for texture resolution testing

**Fig 2:**
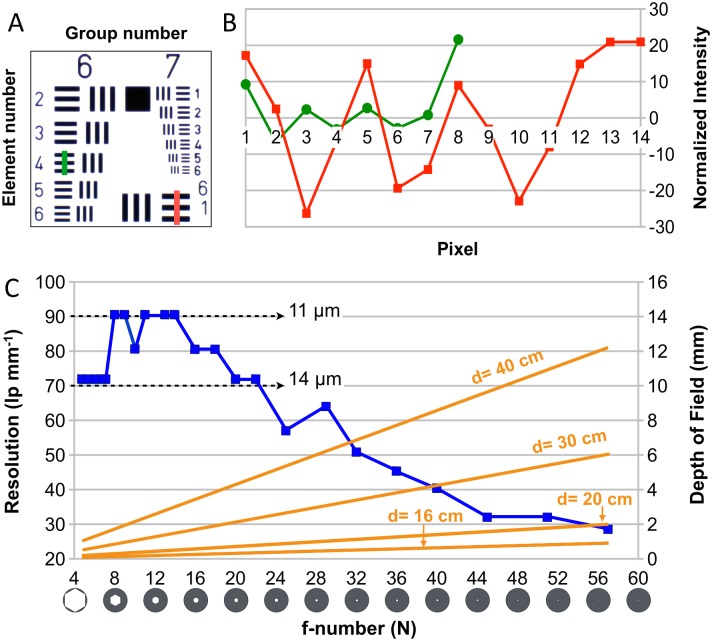
Inferring texture resolution. **A**) The USAF 1951 test target. **B**) Intensity variations along a line of pixels across the test-chart pattern in the 6/4 (green) and 6/1 (red) group/element numbers. The green curve indicates a resolution of at least 11μm. **C**) An increase in f-number (N), *i*.*e*., a decrease of aperture size (as indicated with the camera shutter diagram) causes *(i)* a decrease in resolution (blue line; left vertical axis) because of more intense diffraction but *(ii)* an increase of the depth of field (DOF; right vertical axis, orange lines for four distances between object and camera lens).

A standard USAF 1951 resolution test chart (Fig 2A; Edmund Optics, Barrington, NJ, USA) was used to infer the texture resolution of the system under regular scanning settings and conditions: 105mm lens (Nikon AF-S 105mm f/2.8 VR Micro-NIKKOR), LED illumination, close distance (~16cm, magnification ratio of 1:1) and low ISO (100). The resolving power is given in line pairs per mm (lp/mm) according to:

$$R(lp/mm)=2^{group+\frac{\left(element-1\right)}{6}}.$$ (1)

We then measured intensity variations along a line of pixels across the bars in each group/element numbers of the test chart: a systematic variation between low and high intensities with a period corresponding to the tested group/element numbers demonstrates that the system reaches the corresponding resolution (Fig 2A and 2B). In order to study the effects of blurring by diffraction, this procedure was repeated for various values of the f-number (from f/4.8 to f/57), while adjusting the shutter speed to ensure an exposure value (EV) of 0.

### Preparation of a calibrated target for photometric stereo resolution testing

Dark blue coated polystyrene spheres of 40μm diameter (Phosphorex Inc., Hopkinton, MA, USA) were added to a solution of de-ionised water with 1% Tween-20 until a mass concentration of 20mg/mL was reached. Two different methods were then used to create a monolayer of microspheres:

1. A 20μL droplet of solution was deposited on a plasma cleaned silicon wafer which was then placed on a heated, vibrating plate. The joint effect of thermal motion and vibrations causes the spheres to disperse into a monolayer of tightly packed spheres (after evaporation).
2. A 20μL droplet of solution was deposited on a thin copper filament. A plasma cleaned silicon wafer was then slowly slid beneath the filament at a distance smaller than 1mm. The solution wets the wafer by capillarity and the slow motion of the filament allows a monolayer deposition of packed spheres (after evaporation).

Both methods yielded homogeneous depositions of a monolayer of tightly packed spheres, as confirmed using light or electron microscopy (Fig G Part A in S1 file).

### Analysis of the position repeatability test results

To assess the repeatability of camera position (Fig H in S1 file), the robot was instructed to take 100 close-up pictures at a single pre-defined position, executing several random movements between each photograph and the next. To estimate the bi-dimensional shift between two images (A and B), the following algorithm was used:

1. N (~5000) pixels are randomly picked on image A and their (R,G,B) values are recorded.
2. For X,Y in the range-K to K (~50):
  - Correspond each pixel sampled in point 1 to the pixel on image B with coordinates shifted by X in height and Y in width (*e*.*g*., if a pixel sampled in 1 has coordinates (2014,324) and X = 3,Y = -12, the corresponding pixel in image B will be (2017,312)).
  - For each correspondence assembled in point 2.1, calculate a discrepancy measure given by $\sqrt{{\left(RA-RB\right)}^{2}+{\left(GA-GB\right)}^{2}+{\left(BA-BB\right)}^{2}}$, where (*RA*,*GA*,*BA*) are the (*R*,*G*,*B*) values of the pixel on image A and (*RB*,*GB*,*BB*) the corresponding (shifted) values on image B.
  - Calculate the mean of the N discrepancy measures and store the value as *F*(*X*,*Y*).
3. Determine the minimum of *F* and the corresponding *X*_*min*_,*Y*_*min*_ coordinate. The latter represents a bi-dimensional estimate of the shift between the two images.

Intuitively, the algorithm works by superimposing image A and image B shifted by *X*,*Y* pixels and then selects the best superimposition. For perfect 2D shifts, the value of *F*(*X*_*min*_,*Y*_*min*_) should be 0. However, other effects such as slight variations of the lighting conditions or rotational/axial shifts are ignored in the algorithm’s assumptions and thus will lead to deviations from the ideal situation. The first effect is likely negligible as all the pictures were taken, without refocusing, under the same lighting and exposure conditions. In addition, we found that excluding minima above a certain threshold (*i*.*e*., if *F*(*X*_*min*_,*Y*_*min*_)>*T*, exclude this image pair from the statistics) was an effective way of reducing the influence of rotational/axial shifts as most affected pairs of images had *F*(*X*_*min*_,*Y*_*min*_)>20. We set *T* = 10 for the analysis presented in the main text.

### Analysis of the attenuation of vibrations

For each test scan, the acquired video frames were sorted by camera position and analysed independently. A blur value between 0 (completely sharp) and 1 (completely blurred) was then assigned to each frame by convoluting the picture with a blurring filter and comparing the result with the original image, as described in [31]. Intuitively, pictures that are already blurred will not change considerably after the application of the filter, while sharp images will become blurred. Thus, the dissimilarity between original and artificially blurred pictures will be higher in the second case, and (by convention) lower scores are attributed to such images. Given the camera’s capture rate (30fps) and the time spent by the robot at each position (5s, *i*.*e*., much longer than required to attenuate vibrations), 150 blur values were acquired per position. These were treated as follows:

1. The values were normalised according to *B*_*norm*_ = *B*_*est*_—*B*_*0*_, where *B*_*est*_ is the blur estimated by [31] and *B*_*0*_ is the basal blur level (estimated by the mean of *B*_*est*_ for the last 30 frames, *i*.*e*., from frames experiencing no vibrations). Using normalised blurs is necessary because a position-dependent fraction of each frame will be blurred simply due to out-of-focus effect and not vibrations.
2. The normalised blur values were fitted with the function *Ae* ^-*t/T*^.
3. By capturing a video with the robot fully stopped, we showed that normalised blur values oscillated with an amplitude never exceeding *A*_*th*_ = 0.005. We thus used *A*_*th*_ as the threshold of blurring and estimated attenuation time as *t*_*att*_ = —*T Ln*(*A*_*th*_/*A*) (inset of Fig I in S1 file).
4. If all the normalised blurs in a series were smaller than *A*_*th*_ we considered (conservatively) *t*_*att*_ = 0.1s.

## Results

R^2^OBBIE-3D brings together state-of-the-art robotics, digital camera and LEDs for object 3D geometry and colour texture reconstruction. The system (Fig 1) is based on an industrial six-axis (J1 to J6) robotic arm (M10iA, Fanuc Corporation, Oshino-mura, Japan), a high-resolution DSLR colour camera (D800, Nikon, Tokyo, Japan), an in-house built mechanical extension and illumination basket system with 30 high-intensity LEDs (2100 lumen each), and different stand and suspension configurations. The mechanical interface (Fig A in S1 file), connecting the camera and its illumination basket to the robot flange, was designed to optimise camera scanning envelope and minimise vibrations while keeping the payload as low as possible. Some scanning configurations extend the scanning envelope, in particular the addition of a precision turntable (Fig 1A) commanded directly from the robot controller. The independently-controlled LEDs (Fig B in S1 file) can be used for homogenous illumination (SFM mode) or directional illumination (one LED switched ON at a time—PS mode). Implementation of both the SFM and PS methods allows collecting respectively low- and high-frequency geometry into a combined high-resolution 3D model of the scanned object [32].

For the ‘*static support*’ configuration (Fig 1B), the object to scan is placed on a removable support in front of the robot, allowing for a scanning envelope of about 270° on the horizontal plane for an object (*e*.*g*., an anesthetised lizard) of 30 cm in length. With the ‘*turntable*’ configuration, we can obtain a full 360° scan of larger objects (up to 100cm x 50cm x 50cm). Obviously, these two configurations require to scan the object twice (*e*.*g*., for a lizard, once on the belly and once on the back) to obtain a full model. The alignment of the belly and back scans is straightforward in case the object is rigid whereas it requires specialised non-rigid alignment methods otherwise [33]. For the *‘hanging anesthetised snake’* configuration, the stand and turn-table are removed and the object is suspended into a gutter (*Gs* in Fig 1A) above the robot primary axis (J1), in order to perform a full 360° acquisition in a single scan, provided that the animal is less than 80cm long. On the other hand, the cephalic (anterior) and caudal (posterior) halves of snakes between 80 and 150cm must be scanned separately.

The camera focus and triggering, the LEDs and the turn-table are all electronically and independently controlled from the Robot digital inputs/outputs (IOs). Robot programs are embedded directly inside the robot controller memory such that R^2^OBBIE can work as a full standalone system (Fig C in S1 file), without any manual intervention from start until end of the 3D scanning. In addition, we incorporated a USB/Digital-IO interface (PhidgetInterfaceKit 8/8/8, Phidgets, Calgary, Canada) that allows interaction between the robot and a computer, thus extending the scanning capabilities, such as automatically changing camera settings (*e*.*g*., ISO value, shutter speed, aperture) during a scan. The robotic arm provides high flexibility in programming camera trajectories and can repeat any position with an accuracy of ± 80μm. We use a Nikon D800 camera that incorporates a FX format sensor (36mm x 24mm) with 36.3 effective million pixels (7360x4912) of 4.9μm each, giving an absolute hardware resolution-power limit of 9.8μm (see below) when using a 1:1 reproduction-ratio lens.

We designed the illumination basket (Fig 1D–1F) such that it can accommodate a 105mm (better for close-up) or a 60mm (better for big objects) macro lens. The 30 LEDs provide homogeneous and powerful lighting (with a total of 63000 lumens, *i*.*e*., similar to Xeon flash power) allowing the use of small apertures (*e*.*g*., f/36) and reasonably short exposure time (about 1/100s) at low ISO sensitivity (*e*.*g*., ISO 320) with a distance to target of 40cm (SFM mode). Note that each LED can be turned OFF or ON in less than 0.1s.

### Texture resolution

The resolution of our system is limited by twice the pixel size of the D800’s sensor (2x4.9μm = 9.8μm). However, other factors can significantly influence the resolving power in practical applications: Bayer and anti-aliasing filters, lens quality, f-number, ISO settings, illumination conditions, camera sensor signal-to-noise ratio, image format, etc. Hence, we measured the actual texture resolution of our setup for different values of the f-number (N) using the procedure outlined in the ‘Materials and Methods’ section. Fig 2C (blue line) indicates that the peak resolution of our setup is approximately 90 lp/mm (*i*.*e*., about 11μm given a magnification ratio of 1:1) in the range *N* = f/8 to f/14 while it achieves resolutions between 70 and 90 lp/mm (*i*.*e*., between 11 and 14μm) for all apertures larger than *N* = f/22. Further reductions of aperture cause a gradual drop in resolution, reaching 28.5 lp/mm (about 35μm) at f/57.

These results suggest that *N* values between f/8 and f/14 should be used when resolution is the main concern (as is the case for some PS applications). However, aperture also impacts on the depth of field (DOF) which can be estimated for a symmetrical lens using:

$$DOF=\frac{\left[2sNcf^{2}(s-f)\right]}{\left[f^{4}-N^{2}c^{2}{\left(s-f\right)}^{2}\right]},$$ (2)

where *s* is the distance from the lens to the focus point, *N* is the f-number, *f* is the lens focal length (here, 105mm), and *c* is the so-called ‘circle of confusion’ (the maximum size of the spot at which blurring effects become relevant, *i*.*e*., the double of the sensor pixel size: about 10μm). Fig 2C (orange lines) shows the relation between the DOF and the aperture (*N*) for four typical scanning distances. As reconstruction algorithms (SFM in particular) greatly benefit from pictures with a large portion of their pixels in sharp focus, we found that an aperture of f/32 or f/36 provides a good compromise between resolution and DOF for most applications.

### Geometry resolution

**Fig 3:**
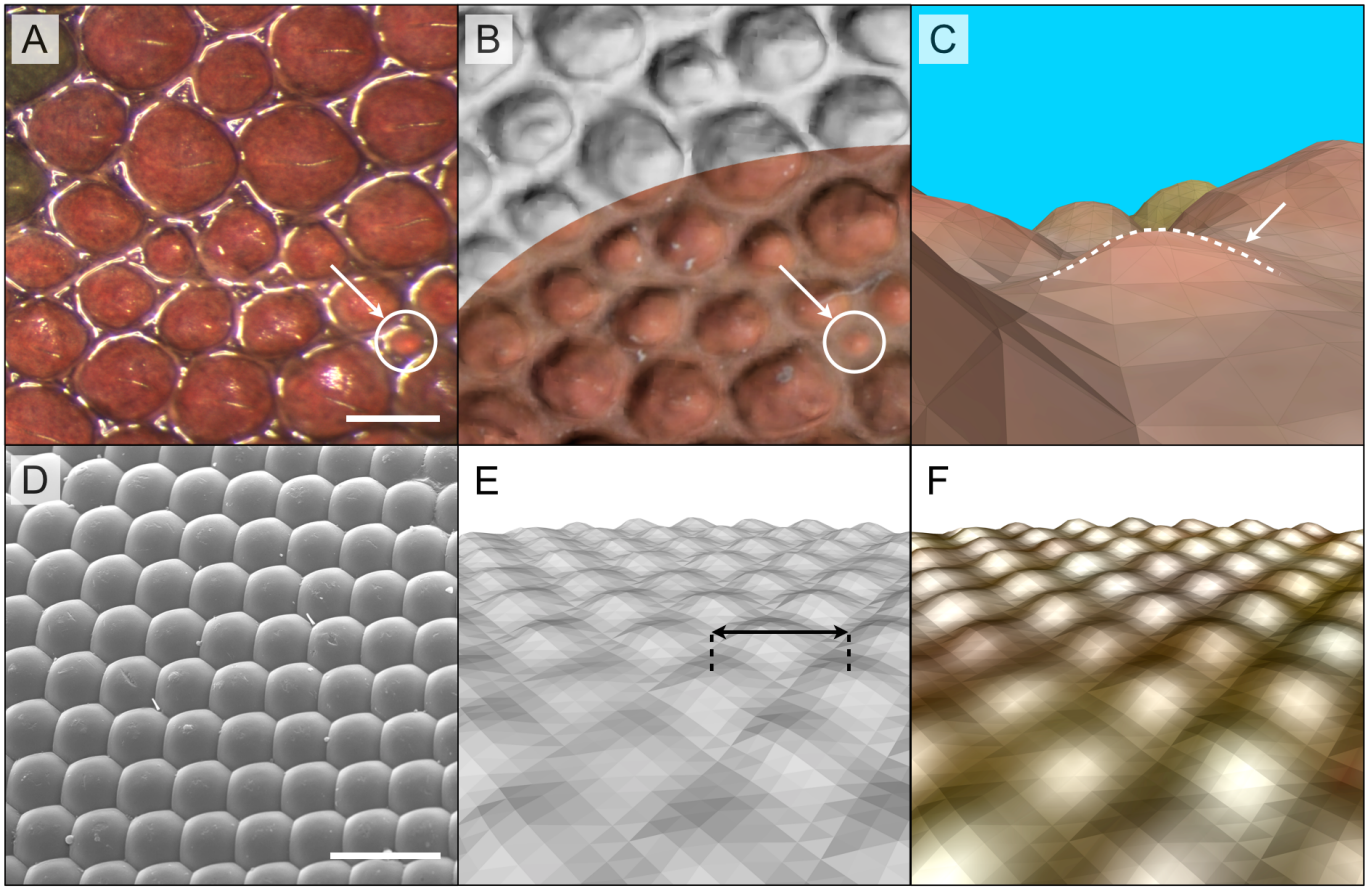
Geometry resolution. **A)** Picture (taken with a stereoscopic microscope) of a skin patch of a juvenile *Phelsuma grandis* gecko. Scale bar: 500μm. **B**) The PMVS reconstruction of the same patch with and without colour texture. **C**) Detail of *B* for the scale indicated with a white arrow. **D)** SEM image of a fly compound eye; Scale bar: 50μm; **E)** Surface geometry and **F)** geometry with colour texture of the same ommatidia as in **D**, reconstructed with Photometric Stereo (double arrows: distance between two ommatidia = 23μm).

For a given magnification, multiview methods such as PMVS have their resolution limited by the efficiency of the feature matching across several images. To estimate the reconstruction resolution of PMVS in our setup, we scanned the dorsal side of a lizard (*Phelsuma grandis*) containing scales of different sizes (Fig 3A). The obtained PMVS reconstruction (Fig 3B and 3C) clearly resolves all scales even without colour texture, including some less than 150μm in diameter.

The second implemented method, photometric stereo, determines surface normals pixel by pixel, making this method particularly well suited for the analysis of fine geometrical details. It could, in principle, reach a resolution of 9.8μm, *i*.*e*., twice the pixel size of the D800’s sensor. However, the actual resolution likely depends on additional specifications of the scanning setup (*e*.*g*., number of light directions) and assumptions of the algorithm such as collinearity of the incident light, and Lambertian (diffuse) reflectance of the scanned object. To quantitatively determine the actual resolution of PS in our setup, we produced a calibrated target (Fig G Part A in S1 file) by depositing a monolayer of 40μm-diameter (nominal value; measured value = 41.1 ± 4.2μm) polystyrene microspheres (Phosphorex Inc., Hopkinton, MA, USA) on a silicon wafer (see ‘Materials and Methods‘). Note that these spheres are shiny, and thus represent a challenging object. We also attempted to reconstruct the micro-geometry of a fly compound eye made of photoreceptor units (ommatidia) arranged with a radial period of 23.2 ± 1.3 μm (N = 193; Fig 3D). The PS scanning and reconstruction of both targets (microspheres, Fig G Parts B and C in S1 file; compound eye, Fig 3E and 3F) conservatively indicate that R^2^OBBIE-3D can easily resolve and reconstruct geometry details smaller than 25μm, even when the scanned object is considerably non-Lambertian. Given that the micro-geometry of the ommatidia are smooth and regular (*i*.*e*., void of obvious noise) and that each ommatidium consists of about 5 pixels, it is likely that the actual geometrical resolution reaches values similar to that of texture resolution demonstrated above, *i*.*e*., 11 to 14μm.

### Repeatability of robot position

The repeatability of robot positions is important for systematic studies as *(i)* it reduces the risk of reconstruction failure due to variation of scanning conditions and *(ii)* allows using robot’s nominal positions to constrain camera calibration during PMVS reconstruction. The specifications of the M10iA robot indicate a repeatability of 80μm for the position of the robot flange. To assess whether a similar value holds for camera position, the following test was performed: using the 105 mm lens, the robot was instructed to take 100 close-up pictures at a single pre-defined position (at 16cm from the target, 1:1 magnification ratio), executing several random movements between each photograph and the next. We then computed the bi-dimensional shifts between each pair of images (see ‘Materials and Methods‘) yielding a distribution of shifts (Fig H in S1 file) with a mean and standard deviation of 0.21 ± 4.80 pixels (1.01 ± 23.52 μm) in the vertical (X) direction and -0.77 ± 9.31 pixels (-3.76 ± 45.63 μm), in the horizontal (Y) direction. Combining both directions yields an average magnitude of shifts of 8.65 ± 5.90 pixels (42.39 ± 28.91μm), and a Pearson correlation coefficient of -0.0998 between shifts in the X and Y directions. This result shows that our system’s positioning is accurate to within a few pixels even at maximum magnification (1:1).

### Attenuation of vibrations

Systematic studies of 3D geometry and colour texture of multiple objects benefit from a fast acquisition process. High scanning speed is even more relevant when working with anesthetised animals. However, the robot’s deceleration as it stops at a position causes vibrations on the camera which, in-turn, can blur the corresponding picture. Whereas the use of fast shutter speeds ensures that low-frequency vibrations do not significantly affect image quality, high-frequency oscillations can be more problematic. To assess the attenuation time of vibrations, we devised the following test: with the camera in video capture mode (30 fps, ~2Mp resolution), we recorded a full lizard scan (58 positions) using five different velocities (10, 25, 50, 75 and 100% of the robot’s maximum velocity). The video frames associated with vibrations were then inputted into an algorithm to estimate blur [31], and post-processed to extract attenuation times (see ‘Materials and Methods‘). As expected, attenuation times increase with the robot’s velocity (linear fit, R^2^ = 0.515) but the mean value remains below 0.6s with standard deviations from 0.5s to 0.9s (Fig I in S1 file). These results indicate that using a robot speed at 30% of the maximum velocity and a waiting time of 2s between reaching a position and triggering the camera conservatively ensure that every picture is not blurred by vibrations of the robot’s arm.

### Examples of 3D geometry and texture reconstructions

**Fig 4:**
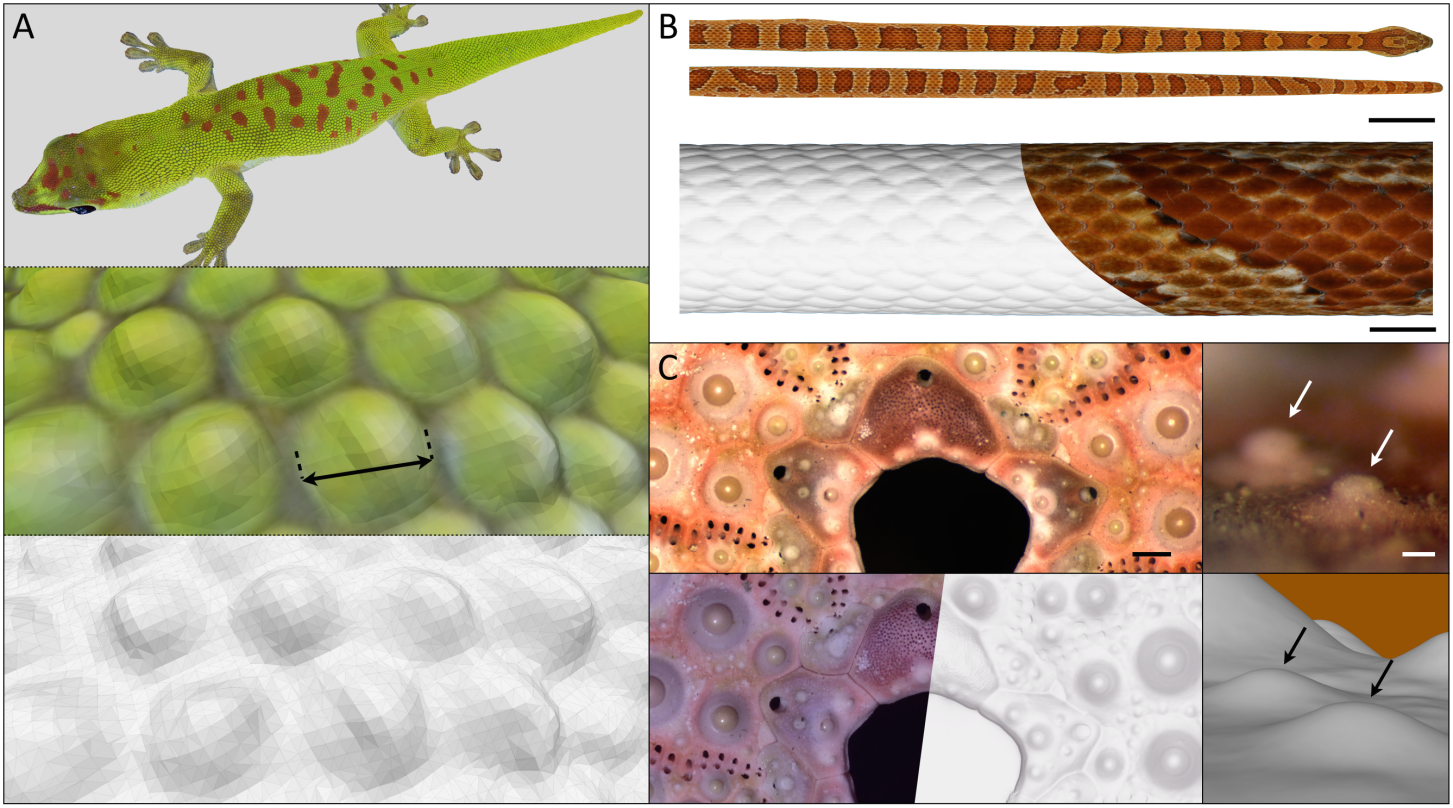
Animal scans performed with R^2^OBBIE. A) An adult day gecko (*Phelsuma grandis*) of 20.5cm (total length) scanned in the ‘static support’ configuration (74 pictures, ~5 minutes of scanning time) and reconstructed with PMVS (~6 hours of reconstruction time); upper panel, overall view; mid panel, close-up with shaded geometry and colour texture (double-headed arrow: 1mm); lower panel, same closeup with geometry only. B) Corn snake (*Pantherophis guttatus*) scanned in the ‘hanging anaesthetised snake’ configuration (424 pictures, ~25 minutes of scanning time) and reconstructed with PMVS (~24 hours of reconstruction time); upper panel, overall view with shaded geometry and colour texture; lower panel, close-up of the shaded geometry with and without colour texture. Scale bars: 40mm (upper panel) and 5mm (lower panel). C) Sea urchin’s (*Echinometra mathaei*) skeleton scanned in the ‘static support’ configuration (30 pictures, ~20 seconds of scanning time) and reconstructed with PS (~20 minutes of reconstruction time); upper-left, photography under a stereoscopic microscope (scale bar: 1mm) without white-balance correction; lower left, reconstructed geometry with and without colour texture (with calibrate white-balance, *i*.*e*., the colour texture is realistic); upper-right and lower right panels, stereoscope image and reconstructed geometry of two small features (arrows) of the specimen (Scale bar: 100μm).

**Fig 5:**
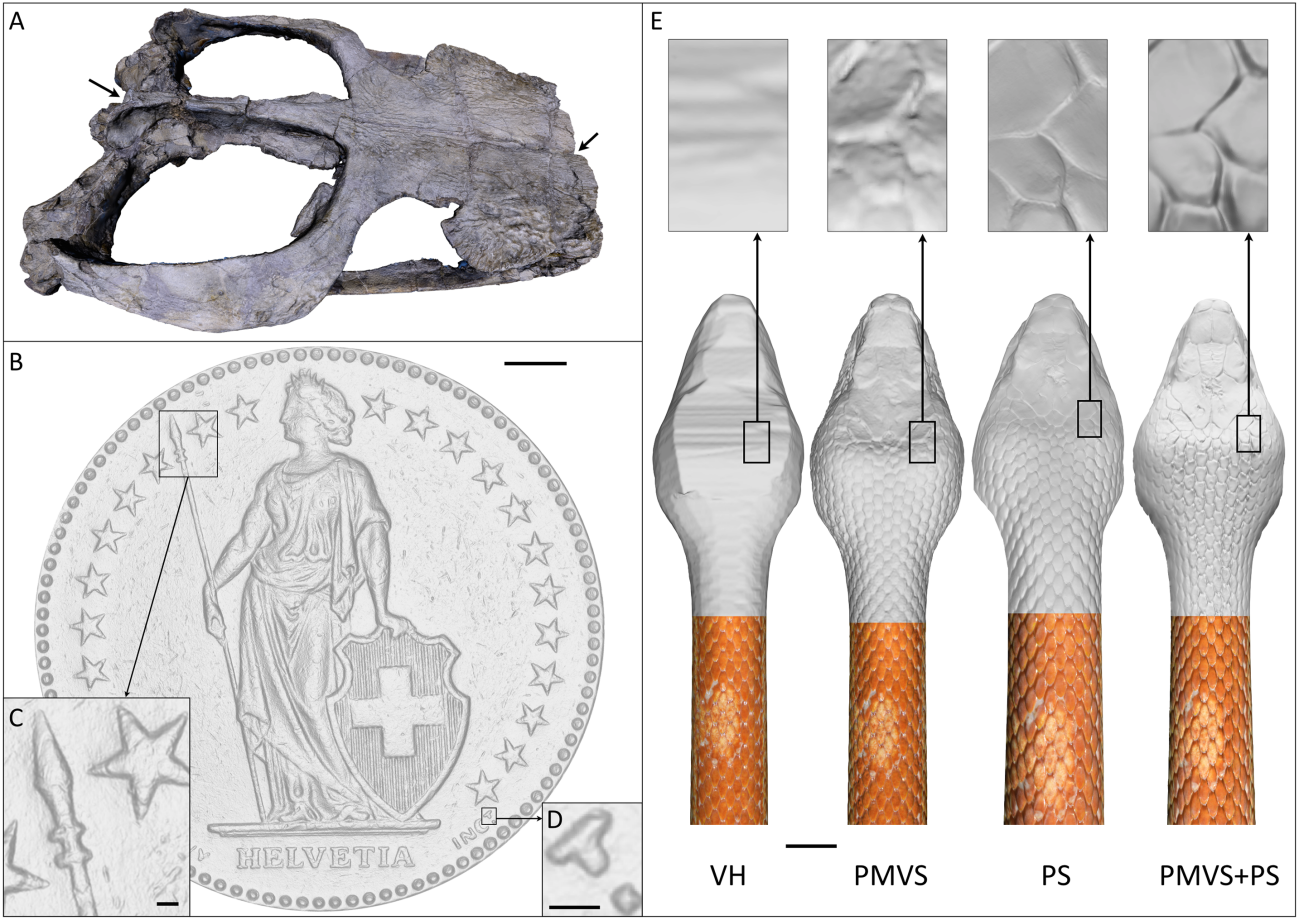
Multiple applications of R^2^OBBIE. A) PMVS reconstruction (~24 hours of reconstruction time) of a 155–161 million-year-old marine crocodylian fossil (*Metriorhynchus superciliosus*; specimen PIMUZ A/III 14 from the Paläontologisches Institut und Museum, Universität Zürich, Switzerland). Multiple scans in the ‘static support’ and ‘turn-table’ configurations were combined for a total of 746 pictures; linear distance between the two arrows = 21.4cm. B) Geometry of a 0.5 Swiss-franc coin scanned in the ‘static support’ configuration (30 pictures, ~20 seconds of scanning time) and reconstructed with PS (~30 minutes of reconstruction time); Scale bar: 2mm. C) and D) Details of B; Scale bars: 200 μm. E) Corn snake (*Pantherophis guttatus*) geometry (and colour texture on bottom half of the mesh) scanned in the ‘hanging anaesthetised snake’ configuration (100 SFM pictures + 30 PS pictures, ~7 minutes of scanning time) and reconstructed with visual hull (VH, ~2 hours of reconstruction time), structure-from-motion (PMVS, ~6 hours of reconstruction time), photometric stereo (PS, ~15 minutes of reconstruction time), and the hybrid mode (PMVS + PS, ~7 hours of total reconstruction time, *i*.*e*., including the PMVS and PS reconstruction times and the combination step). Scale bar: 10 mm. Insets: zoom on geometry.

**Fig 6:**
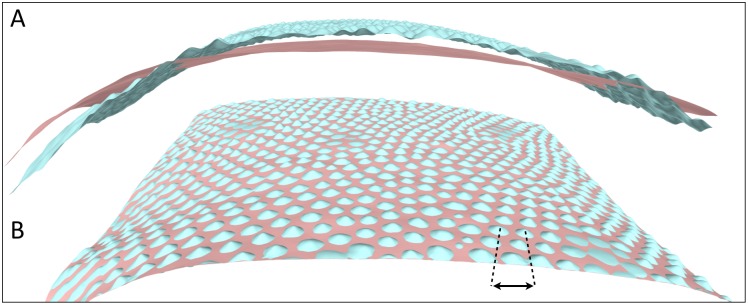
3D reconstruction of a *Phelsuma* gecko skin patch combining SFM and PS. A) The PS reconstruction (blue) produces accurate microgeometry but a biased overall geometry; The PMVS reconstruction (pink) yields an accurate overall geometry and no microgeometry. B) Integration of the low-frequency and high-frequency geometries with a linear optimisation algorithm yields both a correct overall geometry and the body scales microgeometry. Double-headed arrow: the distance between two neighbour scales is about 150 μm.

R^2^OBBIE-3D allows for the fast and robust acquisition of images for 3D reconstruction of basically any object smaller than 100cm x 50cm x 50cm. R^2^OBBIE allows scanning objects in three different modes. First, the SFM mode (capture rate of >15 pictures per minute), used, *e*.*g*., when scanning lizards and snakes for which we investigate the overall skin colour patterns. Fig 4A shows an example of a PMVS reconstruction of an adult day gecko (*Phelsuma grandis*) of ~20cm total length. The 74 pictures used to generate the model were acquired in less than 5 minutes using the ‘static support’ configuration, while the reconstruction step lasted for about 6 hours. An example of a 360° SFM scan and PMVS reconstruction of a corn snake (*Pantherophis guttatus*) is shown in Fig 4B and S2 Movie. The 424 images comprising the scan were acquired in approximately 25 minutes using the ‘hanging anaesthetised snake’ configuration, while the reconstruction step took roughly one day. Note that the scales of this species are slightly reflective and iridescent, making it a challenging scanning target. Second, the PS mode (scanning time of approximately 20s) provides exquisite micro-geometry resolution, but the method generates a bias in low-frequency geometry. These features make this method especially suited for local analyses (*e*.*g*., quantification of local curvature) of very fine geometric details. Fig 4C and S3 Movie show a PS reconstruction of a sea urchin’s (*Echinometra mathaei*) skeleton scanned in the ‘static support’ configuration. Acquiring the 30 pictures took the previously mentioned PS scanning time (20 seconds), while the reconstruction processing time was around 20 minutes. Another PS reconstruction, that of a 0.5 Swiss franc coin (diameter = 18.2mm), is shown in Fig 5B–5D and S4 Movie. The scanning configuration and time were the same as in the sea urchin’s skeleton case, while the reconstruction time was only slightly longer (around 30 minutes). The outputted 3D model shows the very high resolution of the geometry reconstruction despite the strongly reflective nature of the object. Third, the hybrid mode performs a combined SFM+PS run that generates both low-frequency and high-frequency geometric components which are then integrated through a linear optimisation algorithm [32]. Therefore, the hybrid mode of R^2^OBBIE generates a high-resolution and unbiased scan (Fig 6), but is longer to perform (about 20s per position) as it requires capturing 31 pictures for each camera position, *i*.*e*., over 1500 pictures for a typical run of 50 positions. However, note that PS shots do not need to be taken at each SFM camera position, substantially reducing the hybrid scanning time. We use the hybrid mode for analyses involving the geometry of skin appendage such as small body scales. Note that such a hybrid procedure would be impractically tedious to perform manually. Fig 5E shows a comparison between the three approaches mentioned (PMVS, PS and PMVS+PS) for a scan of the rostral part of a corn snake (*Pantherophis guttatus*), comprising 100 SFM and 30 PS (one position) pictures. The scanning and reconstruction times were, respectively, 7 minutes and 7 hours. It is worth noting that each of the three modes can be used for scanning objects either positioned on the scanning platform/turntable (*i*.*e*., ‘static support’ and ‘turntable’ configurations) or suspended above the J1 axis (*i*.*e*., ‘hanging anaesthetised snake’ configuration).

We also used R^2^OBBIE-3D to assess its performances with objects of interest for other research laboratories and museums. Fig 5A and S5 Movie show the reconstruction of a 155–161 million-year-old fossil (head of a *Metriorhynchus superciliosus*, a marine crocodylian from the Upper Jurassic [34]) performed with our system for the Institute and Museum of Paleontology at the University of Zurich, Switzerland. The image data was acquired using 7 SFM scans in both the ‘static support’ and ‘turntable’ configurations. In total, 746 pictures were used in the reconstruction step, which took roughly 24 hours. This example shows that when working with rigid and inanimate objects, our setup can combine multiple scans (to enhance completeness or accuracy) without the need for an alignment software. The model will be 3D printed at its original size for an exhibit where visitors will be allowed to touch objects on display.

Finally, we compared the 3D reconstructions of a corn snake (*Pantherophis guttatus*) performed with the PMVS mode, the PS mode, the hybrid mode (PMVS+PS) and an in-house implemented visual hull algorithm [30] (Fig 5E). The latter approach has recently been used to perform 3D reconstructions of insects on sets of pictures obtained with a DSLR camera, a 5x magnifying lens, a macro rail, and a two-axis turntable [35]. Note that the visual hull reconstruction is much coarser than the PMVS and the PS approaches. For example, in the case of the snake scan, the visual hull reconstruction cannot reliably identify skin scales, making this algorithm unsuitable for applications that require such identification.

## Discussion

Combining state-of-the-art robotics, high-resolution digital cameras, and high-power LED illumination on the hardware side with state-of-the-art SFM and multiview PS reconstruction algorithms on the software side, R^2^OBBIE-3D is able to produce fully-textured 3D models of objects (including animals under anaesthesia), capturing both colour and geometry details from the scale of the meter down to the scale of about 11–25 microns (*i*.*e*., spanning five orders of magnitude) without the use of magnifying lenses. Moreover, the automation of R^2^OBBIE-3D makes the system highly versatile and allows for both practical scanning times and high-throughput analyses.

The digital 3D models generated by R^2^OBBIE-3D provide an accurate quantitative representation of the scanned animal/object that can easily be manipulated using conventional 3D visualisation software (including open source options such as *Meshlab*, [36]; available at *http://meshlab.sourceforge.net*). These models have a wide range of applications such as performing quantitative phenotypic analyses of both surface geometry and colours of specimens, performing simulations of physical/biophysical processes on realistic geometries, or 3D-printing of accurate replicas of animals/objects. Therefore, multiple applications can be found for R^2^OBBIE-3D, not only in the areas of biology/biophysics, but also in, *e*.*g*., biomedical and forensic sciences, biomimicry, museology or reverse engineering.

R^2^OBBIE-3D is highly versatile and generates models of scanned objects with very high geometrical and texture resolution but is not without limitations. Textureless objects cannot be reconstructed with SFM alone because the method requires matching texture features across pictures taken from different camera positions. The problem can be solved by the use of PS (although some bias in the overall geometry will remain, see above). Highly-reflective (*e*.*g*., metallic) and highly-iridescent objects cannot be reliably reconstructed with the SFM approach because, depending on the incident light direction and spatial position(s) of the camera, reflection highlights and/or iridescence will appear on different places on the object, causing serious matching ambiguities. Although, in theory, these objects can also cause serious problems for PS reconstruction, the use of a pre-processing step to remove non-Lambertian reflections effectively gives the algorithm a much higher degree of resilience, as demonstrated by the efficient reconstruction of coins (Fig 5B–5D and S4 Movie). Finally, very sharp features (*e*.*g*., spines) are very difficult to reconstruct with both SFM and PS approaches and transparent objects are equally challenging as their colour texture is, at least partially, a projection from the background. Efficient acquisition of surface geometry of translucent objects could be achieved with R^2^OBBIE-3D by adapting progressive acquisition systems based on dense specularity field information [37].

## Supporting Information

S1 File This PDF file includes nine Supporting Figures as well as a link to 3D models used for the Supporting movies. (PDF)

S1 Movie Scanning with R^2^OBBIE-3D. The movie shows the scanning process in Structure-from-Motion (PMVS) and Shape-from-Shading (PS) modes. See main text for details.
(MP4)

S2 Movie Corn Snake (*Pantherophis guttatus*). Geometry Reconstructed with Structure from Motion. The movie shows the surface geometry and colour texture of a scanned corn snake.
(MP4)

S3 Movie Skeleton of a Sea Urchin. Geometry of a specimen of *Echinometra mathaei* reconstructed with Photometric Stereo. The movie shows the surface geometry and colour texture of a scanned sea urchin skeleton.
(MP4)

S4 Movie Coin of 0.5 Swiss Francs. Geometry Reconstructed with Photometric Stereo. The movie shows the surface geometry of the smallest Swiss coin.
(MP4)

S5 Movie Fossil of Marine Crocodylian. Geometry Reconstructed with Structure from Motion. The movie shows the surface geometry and colour texture of a 155–161 million-year-old fossil (head of a *Metriorhynchus superciliosus*, Upper Jurassic of England). Specimen PIMUZ A/III 14 from the Paläontologisches Institut und Museum, Universität Zürich, Switzerland.
(MP4)
