# Supplementary material for: R2OBBIE-3D, a Fast Robotic High-Resolution System for Quantitative Phenotyping of Surface Geometry and Colour-Texture
Source: PLoS One. 2015 Jun 3;10(6):e0126740. doi: 10.1371/journal.pone.0126740 (PMC4454658; doi:10.1371/journal.pone.0126740)
Supplement: S1 File — (PDF) [file pone.0126740.s001.pdf]

‘Research Article’ ***PLOS ONE (2015)***

R^2OBBIE-3D, a fast robotic high-resolution system for quantitative phenotyping of surface geometry and colour-texture

SUPPORTING INFORMATION FILE S1

António F. Martins[1,2,*], Michel Bessant[1,*], Liana Manukyan[1,2]
& Michel C. Milinkovitch[1,2,#]

[1]Laboratory of Artificial & Natural Evolution (LANE), Department of Genetics & Evolution, University of Geneva, Geneva, Switzerland.

[2]SIB Swiss Institute of Bioinformatics, Geneva, Switzerland.

[*]These authors contributed equally.

[#] Corresponding author:
Michel C. Milinkovitch, Laboratory of Artificial & Natural Evolution (LANE),
Dept. of Genetics & Evolution, University of Geneva,
Sciences III, 30, Quai Ernest-Ansermet, 1211 Geneva 4, Switzerland.
Tel: +41(0)22 379 67 85; Fax: +41(0)22 379 67 95
E-mail: Michel.Milinkovitch@unige.ch

Short title: Colour 3D-geometry high-resolution scanning

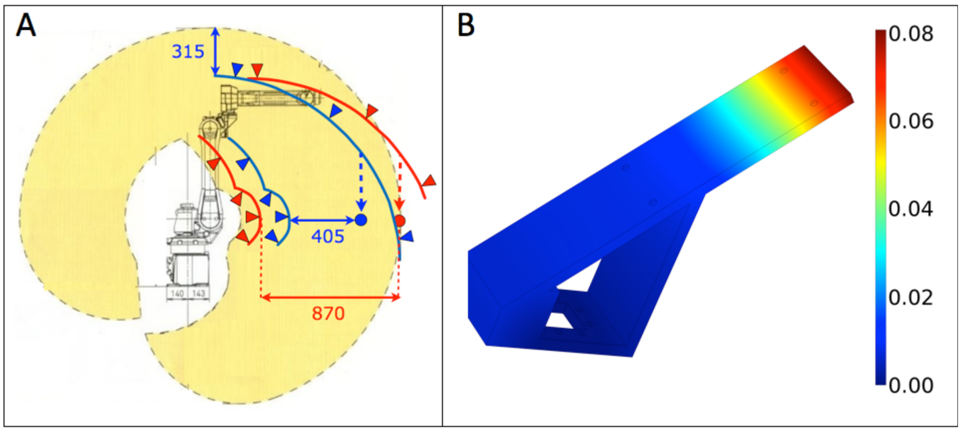

Fig. A. Characteristics of the mechanical extension. (**A**) The working envelope (yellow) is further reduced (blue lines) when the camera box is directly fixed on the robot flange; The mechanical extension enlarges the working envelope (red lines); Working envelopes are considered with the camera looking inside the envelope (arrowheads); With and without the extension, the camera can look down vertically (dashed arrows) towards a target point (blue or red bullet) at respectively 870 and 405 mm from the inside border of the working envelope. (**B**) Deformations of the extension (maximum displacement of 0.08 mm) when the distal end is subjected to a perpendicular force of 75N.

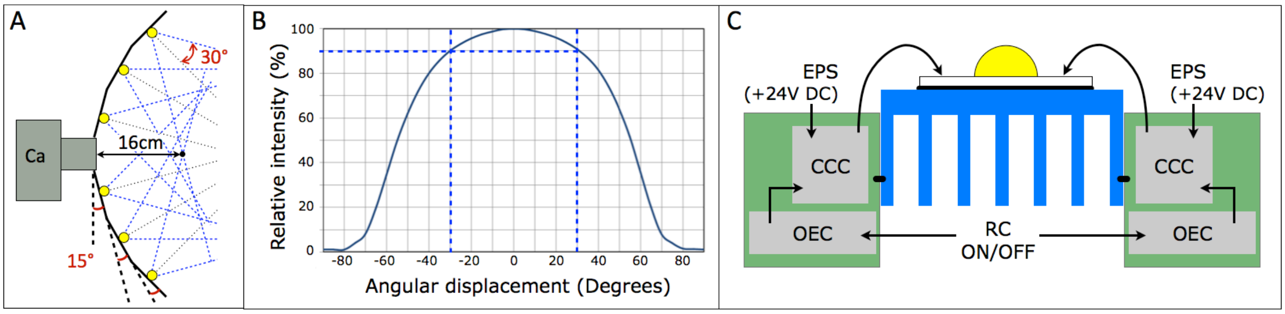

Fig. B. The illumination system. (**A**) The illumination basket with the cone of 90% relative intensity (blue dotted lines) of each LED (yellow) containing the closest possible target object (min focus point at 16cm). (**B**) The LED radiation relative intensity decreases to 90% for an angular displacement of 30°. (**C**) The LED module including a high-power white LED (yellow) fixed on a high-dissipation heat sink (blue) with conductive thermal paste; the LED is powered with two electronic cards (green) each including an ON/OFF electronics control (OEC) connected to the robot controller (RC) ON/OFF command, and a constant-current controller (CCC) connected to an external power supply (EPS).

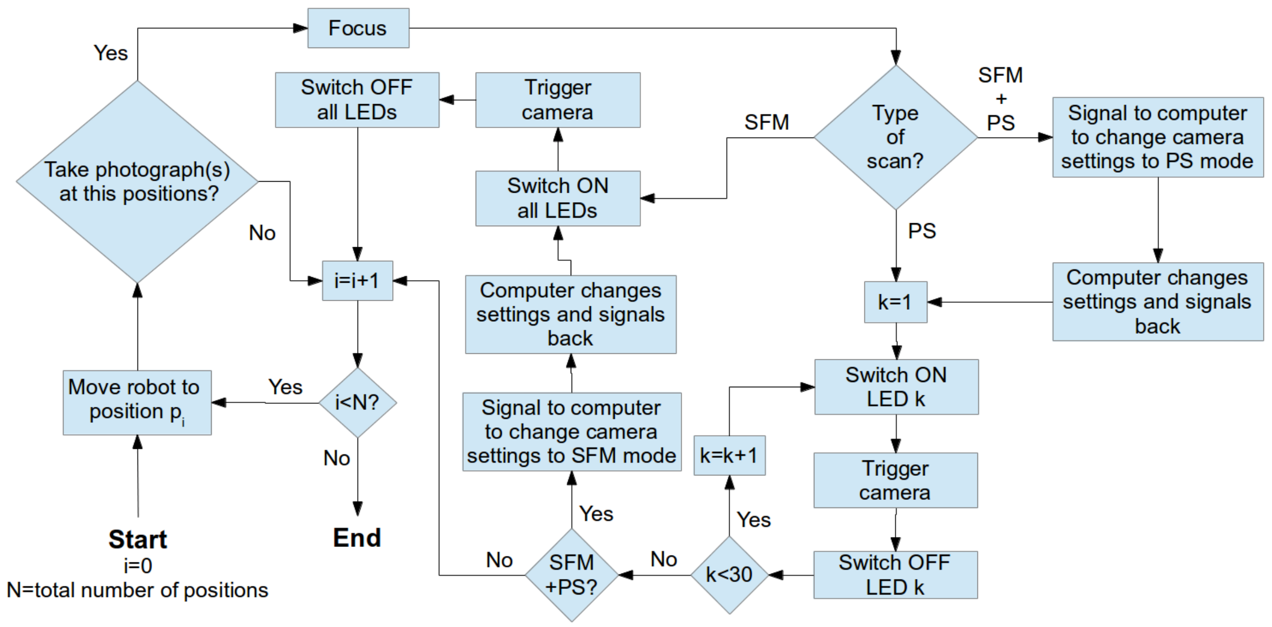

Fig. C. The workflow of a R²OBBIE program. *SFM*, structure from motion; *PS*, photometric stereo, see text for details.

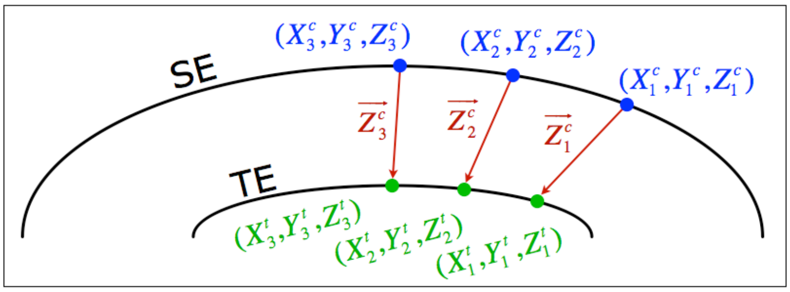

Fig. D. Computing camera positions and orientations. Camera orientation vectors (red) are defined from the camera positions (blue) on the scanning envelope (SE) and from the target positions (green) on the idealised target envelope (TE).

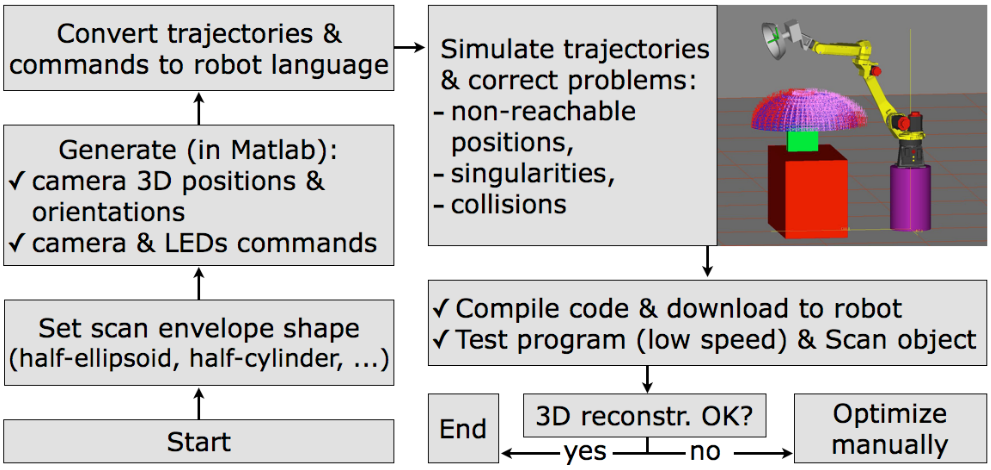

Fig. E. The robot programming flow.

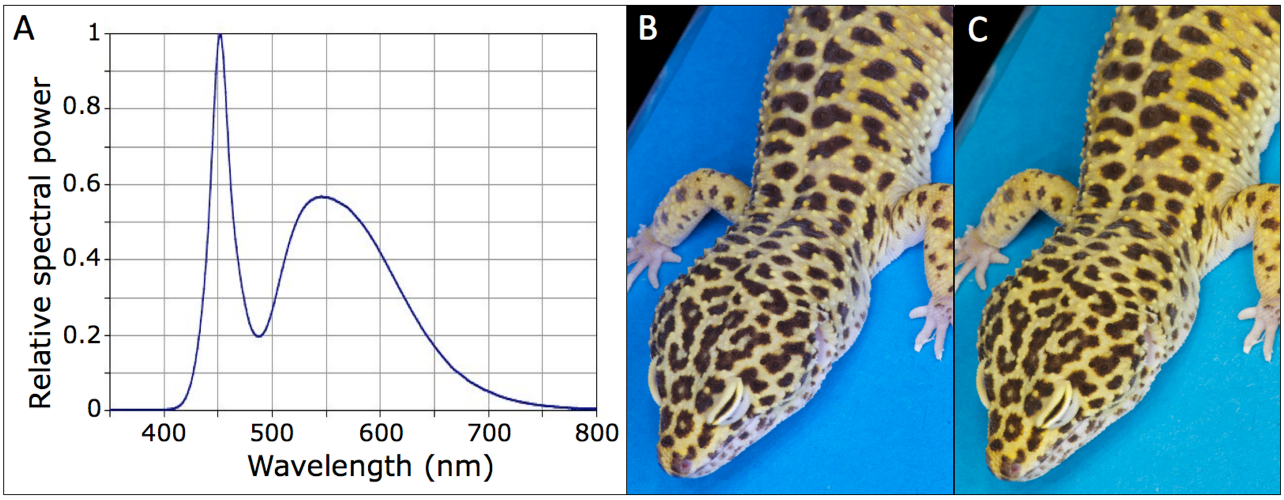

Fig. F. White balance correction. (**A**) White LED spectrum at 5'500K. (**B-C**) Picture of a leopard gecko (*Eublepharis macularius*) before and after white balance calibration.

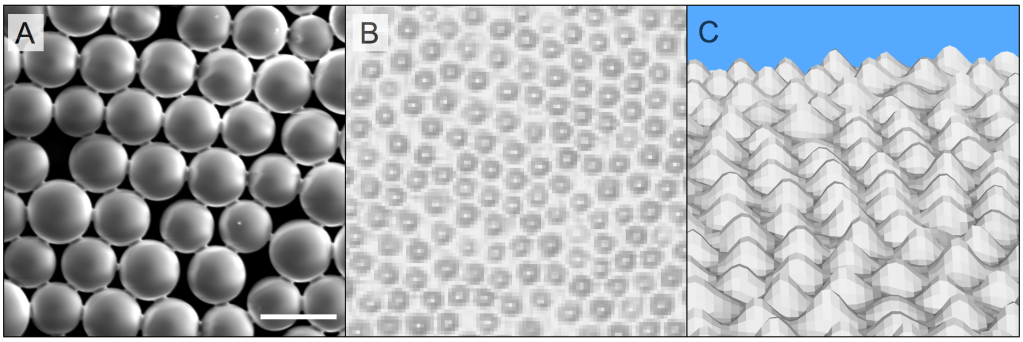

Fig. G. Geometry resolution. (**A**) SEM image of close-packed spheres (diameter = 41.1 ± 4.2µm); Scale bar: 50µm. **(B,C)** Surface of the same spheres as in *D*, reconstructed with Photometric Stereo.

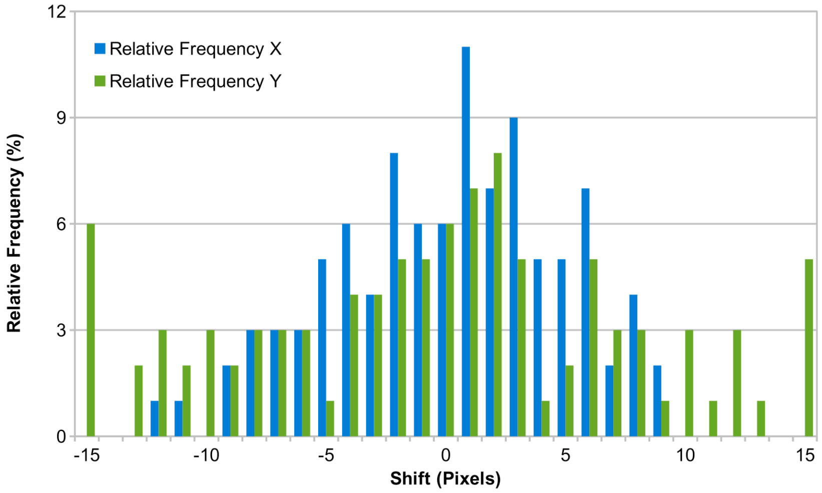

Fig. H. Robot position repeatability. Distributions of image shifts along the X and Y axes due to variation in repeatability.

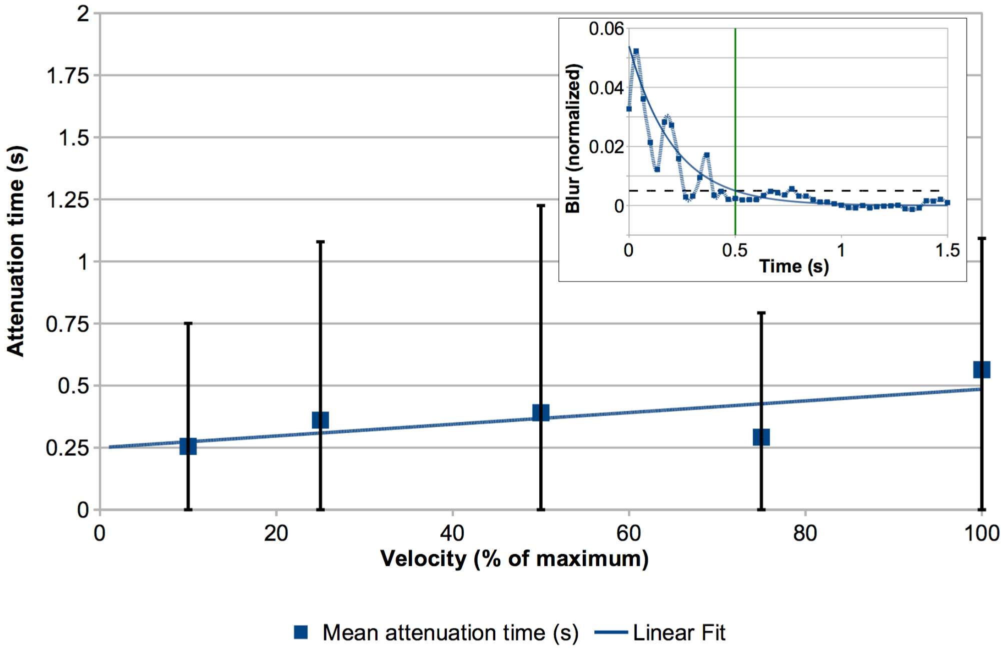

Fig. I. Vibrations attenuation time. Means (squares), standard deviations (vertical bars), and linear fit (blue line; R^2 = 0.515) of vibration attenuation time for various velocities of the robot. Inset: attenuation time (green vertical line) is defined as the time at which the fitted function drops below A_{th} = 0.005 (black dashed line), see details in Materials and Methods.

SUPPORTING MOVIES

- ✓ **S1 Movie *(File: S1_Movie.mp4)* - Scanning with R²OBBIE-3D:** the acquisition process. The movie shows the scanning process in Structure-from-Motion (PMVS) and Shape-from-Shading (PS) modes. See main text for details.
- ✓ **S2 Movie *(File: S2_Movie.mp4)* - Corn Snake (*Pantherophis guttatus*):** Geometry Reconstructed with Structure from Motion. The movie shows the surface geometry and colour texture of a scanned corn snake.
- ✓ **S3 Movie *(File: S3_Movie.mp4)* - Skeleton of a Sea Urchin:** (*Echinometra mathaei*) Geometry Reconstructed with Photometric Stereo. The movie shows the surface geometry and colour texture of a scanned sea urchin skeleton.
- ✓ **S4 Movie *(File: S4_Movie.mp4)* - Coin of 0.5 Swiss Francs:** Geometry Reconstructed with Photometric Stereo. The movie shows the surface geometry of the smallest Swiss coin.
- ✓ **S5 Movie *(File: S5_Movie.mp4)* - Fossil of Marine Crocodylian:** Geometry Reconstructed with Structure from Motion. The movie shows the surface geometry and colour texture of a 155-161 million-year-old fossil (head of a *Metriorhynchus superciliosus*, Upper Jurassic of England). Specimen PIMUZ A/III 14 from the Paläontologisches Institut und Museum, Universität Zürich, Switzerland.

The corresponding model files are available on our laboratory website at http://www.lanevol.org/LANE/R2OBBIE.html).
